# The Impact of Topical Application of Platelet-Rich Fibrin on Graft Survival in Surgeries for Chronic Otitis Media

**DOI:** 10.7759/cureus.53202

**Published:** 2024-01-29

**Authors:** Saurabh Saini, Saquib Reyaz Khan, Masuram Bharath Kumar, Kunal Singh, Priyanshu Pandey

**Affiliations:** 1 Otolaryngology, Varun Arjun Medical College & Rohilkhand Hospital, Shahjahanpur, IND; 2 Clinical Pharmacology, Varun Arjun Medical College & Rohilkhand Hospital, Shahjahanpur, IND

**Keywords:** healing time, graft survival, chronic otitis media (com), tympanoplasty, platelet-rich fibrin (prf)

## Abstract

Objective: This study aims to evaluate the efficacy of platelet-rich fibrin (PRF) application in tympanoplasty procedures for patients with chronic otitis media (COM), assessing its influence on graft survival and healing time.

Methods: In this prospective interventional study, conducted between January 2022 and June 2023, 80 patients diagnosed with COM were enrolled and divided into two groups. Group A underwent standard tympanic membrane repair using temporal fascia grafts (TFG), while Group B received TFG with adjunctive PRF application. The patients were observed and assessed over a 20-week postoperative period.

Results: The study showed a significant enhancement in graft survival rates in Group B (TFG+PRF), with only one residual perforation compared to seven in Group A (TFG alone) at 20 weeks (p=0.02534). Furthermore, Group B patients experienced faster healing, achieving 97.5% graft integrity at 10 and 20 weeks, in contrast to Group A's 87.5%.

Conclusion: The application of PRF in tympanoplasty procedures for COM notably enhances graft stability and expedites the healing process. These findings suggest that PRF can be a valuable adjunct in otolaryngological surgeries, offering potential improvements in patient outcomes and surgical efficacy.

## Introduction

Chronic otitis media (COM) represents a significant global health challenge, impacting a substantial portion of the population with its persistent and debilitating symptoms. This condition is primarily characterized by a perforated tympanic membrane and continuous discharge that persists for over three months, often necessitating surgical intervention for effective management [1]. The standard surgical treatments for COM, which include tympanoplasty, cortical mastoidectomy, and modified radical mastoidectomy, are well-established in clinical practice [2]. However, despite advancements in surgical techniques, the management of COM continues to pose challenges, particularly in terms of graft survival, healing time, and postoperative complications.

The genesis of this research is rooted in the emerging applications of platelet-rich fibrin (PRF) in various surgical fields. PRF, a second-generation platelet concentrate, has garnered attention for its role in wound healing and tissue regeneration [3]. Recent studies have highlighted the potential of PRF to enhance graft survival and expedite healing in various surgical contexts [4]. This promising development in the use of PRF has opened new avenues for its application in otologic surgeries, particularly in the management of COM. However, the specific application of PRF in ear surgeries, especially in the context of COM, still needs to be explored and warrants further investigation.

The rationale for this research is anchored in the potential of PRF to improve surgical outcomes in COM. The high prevalence of COM, coupled with the limitations of current surgical treatments, underscores the urgent need for innovative approaches that can enhance patient outcomes. The application of PRF in COM surgeries could potentially lead to improved graft uptake rates, reduced healing times, and fewer postoperative complications. Such advancements could significantly impact the standard of care for patients suffering from COM, offering a more effective and efficient treatment pathway.

The primary objective of this study is to investigate the impact of topical application of PRF on graft survival in surgeries for COM. By focusing on this innovative approach, the study aims to provide empirical evidence that could potentially revolutionize the standard surgical procedures for treating COM. The exploration of PRF's efficacy in this context is timely and critical in advancing our understanding of its potential benefits in otologic surgeries. This research seeks to contribute to the body of knowledge in otorhinolaryngology and improve surgical outcomes for patients suffering from COM, thereby addressing a significant gap in current medical practice.

## Materials and methods

This prospective interventional study was conducted at the Department of Otorhinolaryngology, Varun Arjun Medical College, Shahjahanpur, Uttar Pradesh, India, from January 2022 to June 2023. Eighty patients diagnosed with COM were enrolled based on their written informed consent and willingness for regular follow-up. The sample size of 80 was calculated for quantifiable variables and equally divided into two groups: Group A consisted of 40 patients undergoing tympanoplasty without PRF, and Group B comprised 40 patients receiving tympanoplasty with autologous PRF.

**Figure 1:**
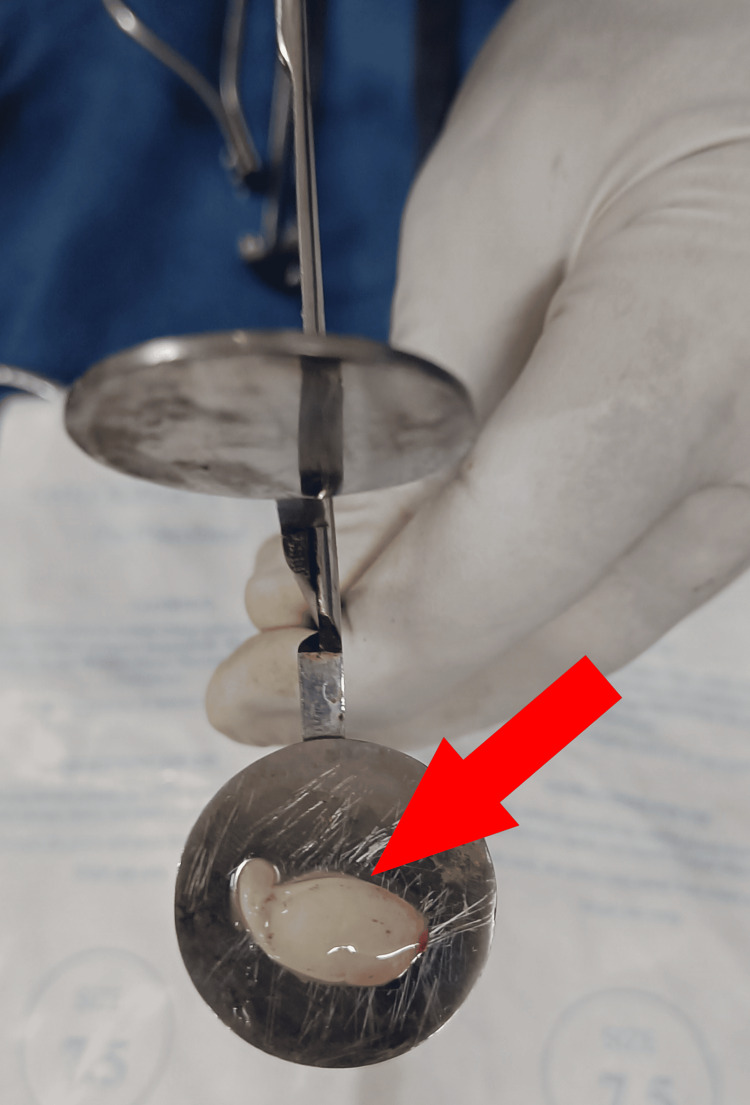
Autologous platelet-rich fibrin obtained by centrifugation.

The study included participants aged between 10 and 60 years who had tympanic membrane perforations for at least 12 weeks without active COM. Individuals below 10 or above 60 years of age, those with chronic illnesses like diabetes mellitus, tuberculosis, bleeding disorders, and cases of COM with complications were excluded from the study. For the preparation of autologous PRF, approximately 5 mL of whole venous blood was collected from each participant's antecubital vein into a sterile vacutainer tube without anticoagulant. Immediate centrifugation at 3000 rpm for 15 minutes yielded three layers: red blood cells, fibrin clot, and cellular plasma. The middle fraction was collected (Figure 1) and transformed into a thin sheet using graft press forceps.

During the surgical procedure, PRF was applied to the edges of the perforation following graft placement. Subsequently, the tympanomeatal flap was repositioned, and the ear canal was packed with gelfoam. In terms of postoperative care, patients received oral antibiotics for the first week and topical antibiotics for the subsequent week. Follow-ups were conducted every 7-15 days for one month, and then monthly for two to three months.

Surgical outcomes were assessed through endoscopic and otoscopic examinations at each patient visit. For statistical analysis, the data were processed using IBM SPSS Statistics for Windows, Version 25 (Released 2017; IBM Corp., Armonk, New York). The chi-square test was employed for categorical variables, and the independent t-test was used for continuous variables. The Fisher exact test was applied in cases where the assumption of independence was not as rigorously met as in categorical tests. Variations in the data were expressed as the mean ± standard deviation (SD).

## Results

In the study, patient demographics and clinical characteristics were similar across both groups (temporal fascia grafts (TFG) and TFG+PRF), with no significant differences in age, gender, perforation side, or perforation type (P-values > 0.05). The average age of patients in both groups was around 32 years, and the distribution of gender, perforation side, and type was comparable between the two groups (Table 1).

**Table 1:** Demographics and clinical characteristics of the patients.

Parameter		Group A	Group B	P-value
Age		32.25 ± 8.61	32.00 ± 8.16	0.904
Gender	Male	15	18	0.649
	Female	25	22	
Perforation side	Right	12 (30%)	17 (42.5%)	0.180
	Left	19 (47.5%)	11 (27.5%)	
	Bilateral	9 (22.5%)	12 (30%)	
Perforation type	Central small	14 (35%)	13 (32.5%)	0.929
	Central medium	11 (27.5%)	12 (30%)	
	Central large	13 (32.5%)	14 (35%)	
	Marginal	2 (5%)	1 (2.5%)	

At 20 weeks post-operation, seven cases had residual perforations in the TFG alone group, compared to one case in the TFG+PRF group. The p-value was 0.02534, indicating statistical significance (Table 2).

**Table 2:** Postoperative graft survival rates.

Time post-operation	Group A	Group B	P-value
4 weeks	36 (95.83%)	39 (97.5%)	0.16585
10 weeks	33 (87.5%)	39 (97.5%)	0.02534
20 weeks	33 (87.5%)	39 (97.5%)	0.02534

For small central perforations, both groups showed 100% graft integrity. In medium and large central perforations, Group B demonstrated higher graft survival rates (medium: 100% vs. 81.8%, large: 92.9% vs. 61.5%), although these differences were not statistically significant (p-values 0.770 and 0.569, respectively) (Table 3).

**Table 3:** Graft perforation and integrity by perforation type.

Perforation type	Group A (perforation/intact graft)	Group B (perforation/intact graft)	Odds ratio	P-value
Central small	14/14	13/13	1.0	1.0
Central medium	11/9	12/12	1.22	0.770
Central large	13/8	14/13	1.50	0.569

## Discussion

The integration of PRF in tympanoplasty for COM represents a novel approach in otologic surgery, potentially addressing some limitations of traditional surgical methods. Our study indicates an improvement in graft survival rates with PRF, particularly at 10 and 20 weeks post-operation. This finding is in line with burgeoning research on PRF's role in tissue regeneration and wound healing, as demonstrated in various surgical fields [5-7].

However, the study's limitations, including its sample size and lack of control for variables such as surgical technique variations and postoperative care, necessitate a cautious interpretation of the results. These limitations highlight the need for more extensive, randomized controlled trials to validate the efficacy of PRF in tympanoplasty and to establish standardized protocols for its use [8,9].

The study's findings suggest that the size or type of tympanic membrane perforation does not significantly influence PRF's effectiveness. This observation aligns with the understanding of PRF's biological properties, which are thought to be the primary contributors to its beneficial effects, irrespective of the physical characteristics of the perforation [10,11].

The role of PRF in enhancing graft survival could be attributed to its ability to release molecules like von Willebrand factor, P-selectin, fibronectin, vascular endothelial growth factor (VEGF), platelet-derived endothelial growth factor (PDEGF), vitronectin, and fibrinogen that promote tissue regeneration and angiogenesis, as well as its potential to modulate inflammatory responses, which are crucial in the healing process [12,13]. These properties of PRF may explain the improved outcomes observed in the TFG+PRF group in our study.

Despite these promising findings, it is imperative to approach the results with caution. The positive impact of PRF observed in this study is a preliminary step in understanding its role in tympanoplasty. Further research is needed to explore the long-term effects of PRF on patient outcomes and to investigate its mechanism of action in the context of tympanic membrane repair [14].

## Conclusions

The addition of PRF in tympanoplasty for COM appears to enhance graft survival rates and expedite the healing process, particularly in the medium-term postoperative period. However, these findings should be interpreted considering the study's limitations. Future research should focus on larger-scale studies to confirm these results and to further elucidate the role of PRF in otologic surgery.
